# Patient Outcomes following Subarachnoid Hemorrhage between the Medical Center and Regional Hospital: Whether All Patients Should Be Transferred to Medical Centers

**DOI:** 10.1155/2014/927803

**Published:** 2014-07-14

**Authors:** Tsung-Ying Lin, Chieh Hsin Wu, Wei-Che Lee, Chao-Wen Chen, Liang-Chi Kuo, Shiuh-Lin Huang, Hsing-Lin Lin, Chih-Lung Lin

**Affiliations:** ^1^Division of Trauma, Department of Surgery, Kaohsiung Medical University Hospital, Kaohsiung Medical University, 100 Tzyou 1st Road, Kaohsiung 807, Taiwan; ^2^Department of Emergency Medicine, Kaohsiung Medical University Hospital, Kaohsiung Medical University, Kaohsiung 807, Taiwan; ^3^Division of Neurosurgery, Department of Surgery, Kaohsiung Medical University Hospital, Kaohsiung Medical University, Kaohsiung 807, Taiwan; ^4^Graduate Institute of Medicine, College of Medicine, Kaohsiung Medical University, Kaohsiung 807, Taiwan; ^5^Department of Emergency Medicine, Faculty of Medicine, College of Medicine, Kaohsiung Medical University, Kaohsiung 807, Taiwan

## Abstract

Subarachnoid hemorrhage (SAH) is a critical illness that may result in patient mortality or morbidity. In this study, we investigated the outcomes of patients treated in medical center and nonmedical center hospitals and the relationship between such outcomes and hospital and surgeon volume. Patient data were abstracted from the National Health Insurance Research Database of Taiwan in the Longitudinal Health Insurance Database 2000, which contains all claims data of 1 million beneficiaries randomly selected in 2000. The International Classification of Diseases, Ninth Revision, subarachnoid hemorrhage (430) was used for the inclusion criteria. We identified 355 patients between 11 and 87 years of age who had subarachnoid hemorrhage. Among them, 32.4% (115/355) were men. The median Charlson comorbidity index (CCI) score was 1.3 (SD ± 0.6). Unadjusted logistic regression analysis demonstrated that low mortality was associated with high hospital volume (OR = 3.21; 95% CI: 1.18–8.77). In this study, we found no statistical significances of mortality, LOS, and total charges between medical centers and nonmedical center hospitals. Patient mortality was associated with hospital volume. Nonmedical center hospitals could achieve resource use and outcomes similar to those of medical centers with sufficient volume.

## 1. Introduction

The annual subarachnoid hemorrhage incidence is 7–20/100 000/y [1–5]. Mortality rates of this devastating disease range from 32% to 50% [3, 6–8]. The overall prognosis of patients with subarachnoid hemorrhage remains poor with nearly half of the survivors diagnosed with sequelae [8, 9], which is associated with substantial financial burdens on the healthcare system. Acute subarachnoid hemorrhage patients are generally sent to the nearest hospitals, although some of them may request a transfer to medical centers. Because numerous regions lack medical centers, patients typically remain at nonmedical center hospitals to receive treatment.

Most medical centers in Taiwan are training hospitals, and young trainees and residents remain in a hospital for several years, until their abilities and experience are sufficient for transferring to other hospitals. Therefore, compared with nonmedical center hospitals, whether the outcomes and medical expenses in medical centers are more favorable is uncertain. Standardized process-of-care measures might play a role in optimizing quality and efficiency, regardless of hospital or surgeon volume [10].

Acute subarachnoid hemorrhage is a disease that can be fatal if it occurs abruptly. Clinical decision-making and policy-making for subarachnoid hemorrhage are challenging and require effective planning and medical care. In this study, we used nationwide population-based data on all hospitalizations for subarachnoid hemorrhage between 2000 and 2009, from the Taiwan National Health Insurance Research Database (NHIRD), to analyze the associations between outcome and hospital level. The underlying assumption is that medical centers may achieve enhanced outcome and low cost in treating subarachnoid hemorrhage. The second aim of this population-based study was to explore the predictors of hospital resource use and mortality rates in a population of patients who had acute subarachnoid hemorrhage.

## 2. Materials and Methods

### 2.1. Data Source

The National Health Insurance (NHI) program, established in March 1995, is the only public insurance system for the entire population of Taiwan and is a universal healthcare system covering 99% of the country's population of 23 million. Patient data were abstracted from a subdataset of the NHIRD in the Longitudinal Health Insurance Database 2000, which contains all claims data (from 1996 to 2009) of 1 million beneficiaries randomly selected in 2000. Between the sample groups and all enrollees, no significant difference existed in age, gender, or health care costs. The encrypted secondary database contains patient-level demographic and administrative information including sex, birthdates, dates of admission and discharge, hospital level of the institutions providing services, the International Classification of Diseases, Ninth Revision, Clinical Modification (ICD-9-CM) diagnosis (up to 5) and procedure (up to 5) codes, status of patient discharge (recovered, died, or transferred out), and hospital charges of all medical expenses. This program provides a highly reliable database for researchers. The study protocol was approved by the Institutional Review Board of Kaohsiung Medical University.

### 2.2. Study Sample

All patients included in the study had been discharged from a hospital included in the NHIRD during the 10-year period between 2001 and 2009. International Classification of Diseases, Ninth Revision, Clinical Modification classification code subarachnoid hemorrhage (430) was used for the inclusion criteria. The exclusion criteria included ICD-9-CM 800.0–801.9, 803.0–804.9, 850.0–854.1, and 873.0–873.9 (head injury).

### 2.3. Variables

In-hospital mortality, total charges during hospitalization, and hospital LOS were used as the outcome variables of this study. Patient age, sex, and the CCI score were used as covariates. Hospital-level covariates used in adjustment included geographical region (Northern, Central, and Southern Taipei and Eastern Kao-Ping) and accreditation level (academic medical center, regional, and district). We further categorized an “academic medical center” as a medical hospital and a “regional or district hospital” as a nonmedical center hospital.

The mortality rate was defined as being within 30 days of hospital admission, as suggested by the Centers for Medicare and Medicaid Services. Because the NHI in Taiwan is a single-payer health program, the only reason for being withdrawn from NHI coverage within 30 days of hospital admission would be that the patient had expired (the other 2 conditions for withdrawal from the NHI coverage, being incarcerated for over 2 month or disappearing for over 6 month, would not be possible reasons for withdrawal within 30 days of hospital admission) [11].

Because the hospital and surgeon volumes in our data did not constitute a normal distribution and the volume categories developed by Peterson et al. [12] may not optimally characterize the distribution of our study, we simply divided the distribution of the volumes during the study period into approximately 2, according to hospital volume (less than 30 and more than 30) and surgeon volume (less than 2 and more than 2). The lowest volume of surgeons and hospitals served as the reference group.

### 2.4. Statistical Analysis

The patient characteristics were analyzed between the medical center hospital and the nonmedical center hospital to determine differences in age, sex, CCI, and surgical clipping rate. After confirming that no differences in patient characteristics existed between the hospital levels, we further analyzed the outcomes regarding mortality, medical expenditure, and hospital LOS. The mortality of these patients was compared for differences in their demographic characteristics and hospital level by using bivariate analysis. Categorical variables were compared using the *χ* ^2^ or a Mann-Whitney 2-independent-sample test. Unadjusted and adjusted logistic regression analyses were used to estimate the odds ratio (OR) and its 95% confidence interval (CI) between mortality and age, sex, CCI, hospital levels, and hospital and surgeon volume. A value of *P* < .05 was considered statistically significant. All statistical calculations were performed using the Statistical Package for Social Sciences for Windows (SPSS for Windows 19.0).

## 3. Results

### 3.1. Patient Characteristics

We identified 355 patients between 11 and 87 years of age who had acute subarachnoid hemorrhage. Among them, 32.4% (115/355) were men. The median CCI score was 1.3 ± 0.6. Of the patients included in the study, 25 died while being in the hospital (7.0%), LOS was 23.1 ± 13.2 days, and the total admission charge was NT$365848 ± NT$221534 (US$1.00 = NT$30.27 in 2011) (Table 1).

### 3.2. Patient Characteristics between Hospital Levels

In the univariable analysis, fewer patients were treated in nonmedical centers than in medical centers (101 versus 254). The 2-independent-sample test analysis and the Pearson *χ* ^2^ test showed no significant differences between medical and nonmedical center hospitals in patient characteristics (sex, age, surgical clipping rate, and CCI). Outcomes including in-hospital mortality, LOS, and total admission charges between the hospital levels had no statistical differences (Table 2). One hundred percent of the nonmedical hospitals had a volume exceeding 30 and 40.2% of medical centers had a volume exceeding 30. Surgeon volume of nonmedical centers was 36.6% more than 2 and 46.5% of medical centers had a surgeon volume exceeding 2 during the study period.

### 3.3. Outcomes between Hospital Levels

The unadjusted and adjusted logistic regression analyses (enter model) were used to estimate in-hospital mortality. Unadjusted logistic regression analysis demonstrated that low mortality was associated with hospital volume (OR = 3.21; 95% CI: 1.18–8.77). After we adjusted for patient's sex, surgeon volume, hospital level (medical center versus nonmedical center hospital), and CCI, significant associations existed in hospital volume (*P* = .024; OR = 0.277; 95% CI: 0.091–0.842) with the 30-day mortality (Table 3).

We observed a significant association between total charges and LOS (*P* < .001) after adjusting for patient age, sex, mortality, and hospital level using linear regression analyses. Medical centers were associated with a trend toward increased total charges, although this was not statistically significant after adjustment. After we adjusted for patient age, sex, mortality, and hospital level by using linear regression analyses, LOS was not associated with any variants.

## 4. Discussion

We used nationwide population-based data to evaluate the difference between mortality and LOS, as well as the total charges of acute subarachnoid hemorrhage treated between medical centers and nonmedical center hospitals (regional and district). We observed no statistical significance in mortality, LOS, or total charges between medical centers and nonmedical center hospitals. However, patient mortality was associated with hospital volume.

Acute subarachnoid hemorrhage is a complex disease that is associated with high risk of mortality and morbidity. Medical centers have more medical resources and facilities than other levels of hospitals, including sophisticated intensive care units, live support equipment, and specialist personnel. Therefore, centralized patients might have beneficial outcomes. However, in this study, we found that patient mortality was associated with hospital volume rather than hospital level. The outcome of acute subarachnoid hemorrhage treated in nonmedical center hospitals yielded results similar to those of medical centers. In Taiwan, only board-certified neurosurgeons can perform acute subarachnoid hemorrhage surgery and neurosurgeons should be trained in medical centers for more than 7 years before obtaining board certification after full training for a national examination. Thus, neurosurgeons practicing in regional or district hospitals have full capability and clinical experience for patient care and surgical skills.

The mortality rate for patients with acute subarachnoid hemorrhage was 7% in this study, similar to the 8.8%–48.5% in the United States, and 9.6% in the Japan study that investigated the relationships between case volume and outcome [13–18]. Numerous studies have shown the relationships between hospital volume and outcome in cerebral aneurysm clipping [19, 20]. Leake et al. evaluated the US National Inpatient Sample for the period 2001–2008 for outcomes and trends in patient admissions for treating subarachnoid hemorrhage at high- and low-volume centers [19], concluding that the treatment of ruptured cerebral aneurysms increasingly occurs at high-volume centers in the United States. They observed enhanced outcomes associated with treating these lesions at high-volume centers [19]. However, another study conducted in Japan did not favor the same conclusion [20]. Hattori et al. analyzed a nationwide study to investigate the relationships between case volume and outcome in cerebral aneurysm clipping surgery performed in 2003. A total of 11974 clipping procedures were included in the report. The final data showed that a greater case volume did not correlate with a favorable outcome, which indicated that higher volume in medical centers than in nonmedical center hospitals may not influence the outcomes [20]. In our study, we found that patient mortality was associated with hospital volumes rather than hospital level. Cross et al. also found that high-volume subarachnoid hemorrhage treatment centers might improve overall survival [21]. We suggest that volume is a crucial factor because of medical centers having more patients; however, a standardized quality of care in nonmedical center hospitals could improve outcomes if their neurosurgeons undergo effective training.

In this study, we found no significant reduction of LOS, mortality rate, and total charges between the 2 hospital levels. One of the reasons may be that patients with more complications were transferred to medical centers. However, the Taiwan health care system allows patients free access to any hospital of their choice; thus, medical centers attract difficult cases and have numerous less severe patients. In addition, subarachnoid hemorrhage patients might not be regionalized before transferring to a hospital; thus, patients are generally sent to the nearest hospital. This practice leaves little room for deliberate patterns of selective referral to either specific hospitals or attending physicians. Moreover, patients sent to nonmedical center hospitals might not be able to be transferred because of critical conditions. We observed no differences in medical expenditure between the hospital levels. To manage a higher medical workload, medical centers typically employ more physicians and nurses than nonmedical center hospitals do. The staff-to-patient ratio may often be higher in larger hospitals. Subspecialists, including cerebrovascular neurosurgeons, interventional neuroradiologists, neuroanesthesiologists, and neurointensivists, are also more likely to be included in the staff. Patients admitted to medical centers may have more routine checkups and exams following visits with specialists. Therefore, although medical centers might have more treatment experience and might reduce certain aspects of medical expenditure during management, they spend more on expansion, which eventually makes no difference with nonmedical center hospitals.

Surgical skills improve with increased experience, but they are also affected by surgeon training. In Taiwan, the Taiwan Neurosurgical Society conducts neurosurgical specialist training, which has established high standards for neurological surgeries, including cerebral aneurysm clipping. The neurosurgeon should be trained in medical centers for more than 7 years and become board certified after a national examination. In Taiwan, neurosurgeons practicing at nonmedical center hospitals should have more confidence in treating patients without transfer. Therefore, by providing an effective training system, the performance of nonmedical center hospitals in treating subarachnoid hemorrhage might not be inferior to that of medical centers if neurosurgeons demonstrate sufficient confidence.

*Limitations*. Despite the strengths of our study using a national database, the findings must be interpreted with caution because of the following limitations. The NHIRD contains a complete representation of all cases admitted to all hospitals of Taiwan. However, this study is a retrospective review of the NHIRD data; therefore, there is significant potential for selection bias and uncontrollable factors that could influence the outcomes of medical center and nonmedical center hospitals. We attempted to address this by using logistical analysis to adjust for several patient-specific and hospital-specific factors, and the purpose of this study was not to look for those factors. A further limitation of our study is the lack of data in the NHIRD on aneurysm-specific characteristics, such as aneurysm location and size, and clinical data, including the severity of SAH and patient's Glasgow Coma Scale score. However, in our study, the CCI exhibited no difference between the hospital levels, which may present no statistical difference in patient comorbidity between hospital levels. Certain information, such as times from bleeding to surgery, rebleeding, and the use of lumbar drainage, was not recorded in the database. Therefore, we cannot provide these outcomes in this study. Another limitation of this study is that we relied exclusively on claims data, which may result in potential disease classification bias. However, we are confident in assuming that the patient characteristics were similar at the hospital levels, which may decrease the bias in our results.

## 5. Conclusion

We observed no statistically significant measures of mortality, LOS, or total charges between medical centers and nonmedical center hospitals of patients with acute subarachnoid hemorrhage. Patient mortality was associated with hospital volume. Nonmedical center hospitals with sufficient hospital volumes could achieve similar resource use and outcomes as medical centers in treating subarachnoid hemorrhage.

## Figures and Tables

**Table 1:** Patient demographics of study population and overall hospital characteristics for brain aneurysm surgery^a^.

Characteristic	Finding
Patients, total number	355
Male sex	115/355 (32.4%)
Age, median (SD), y	57.2 (14.8)
CCI, median (SD)	1.3 (0.6)
Hospital characteristics	
30-day mortality	25/355 (7.0%)
LOS, median (SD), d	23.1 (13.2)
Total charges, median (SD), $NTD	365,848 (221,534)

LOS: length of stay; CCI: Charlson index comorbidity score.

^a^Unless indicated otherwise, data are reported as number of patients in the relevant category/number of patients possible in the category (percentage). US$1.00 = NT$30.27 in 2011.

**Table 2:** Univariable analysis of patient demographics and hospital characteristics for brain surgery of aneurysm stratified by hospital status^a^.

Characteristic	Hospitals of other levels (*n* = 101)	Medical center (*n* = 254)	*P *
Female (%)	68/101 (67.3)	172/254 (67.7)	0.944^b^
Age, median (SD), y	58.4 (13.8)	56.8 (15.2)	0.355^c^
CCI, median (SD)	1.3 ± 0.5	1.3 ± 0.7	0.814^c^
Surgical clipping (%)	58/101/(57.4)	143/254 (56.3)	0.847^b^
Hospital characteristics			
In-hospital mortality (%)	8/101 (7.9)	17/254 (6.7)	0.683^b^
LOS, median (SD), d	23.2 (12.5)	23.2 (13.5)	0.991^c^
Total charges, median (SD), $NTD	356,637 ± 174,594	369,511 ± 237,851	0.622^c^

LOS: length of stay; CCI: Charlson index comorbidity score.

^a^Unless indicated otherwise, data are reported as number of patients in the relevant category/number of patients possible in the category (percentage). ^b^Pearson *χ* ^2^ test.

^c^Two-independent-sample test (Mann-Whitney *U*). US$1.00 = NT$30.27 in 2011.

**Table 3:** Independent risk factors associated with mortality.

Factor	Odds ratio	*P* value	95% confidence interval
Hospital volume	0.277	0.024	0.091–0.842
Surgeon volume	1.700	0.277	0.653–4.423
Hospital levels	0.648	0.374	0.249–1.686
Gender	0.921	0.864	0.362–2.348
Age	1.028	0.094	0.995–1.061
CCI	1.453	0.135	0.890–2.374

The risk factors included in the logistic regression model were surgeon volume, hospital volume, hospital levels, gender, age, and CCI.
